# Chemical features mining provides new descriptive structure-odor relationships

**DOI:** 10.1371/journal.pcbi.1006945

**Published:** 2019-04-25

**Authors:** Carmen C. Licon, Guillaume Bosc, Mohammed Sabri, Marylou Mantel, Arnaud Fournel, Caroline Bushdid, Jerome Golebiowski, Celine Robardet, Marc Plantevit, Mehdi Kaytoue, Moustafa Bensafi

**Affiliations:** 1 Lyon Neuroscience Research Center, University Lyon, CNRS UMR5292, France; 2 Food Science and Nutrition Department, California State University, Fresno, California, United States of America; 3 INSA Lyon, CNRS, LIRIS UMR5205, France; 4 Infologic, Bourg-lès-Valence, France; 5 Ecole Nationale Polytechnique d’Oran—Maurice Audin, Département de Mathématiques et Informatique, Oran, Algérie; 6 Institute of Chemistry of Nice, UMR CNRS 7272, Université Côte d’Azur, Nice, France; 7 Department of Brain & Cognitive Sciences, DGIST, Daegu, Republic of Korea; 8 Université Lyon 1, CNRS, LIRIS UMR5205, France; Rutgers University, UNITED STATES

## Abstract

An important goal in researching the biology of olfaction is to link the perception of smells to the chemistry of odorants. In other words, why do some odorants smell like fruits and others like flowers? While the so-called stimulus-percept issue was resolved in the field of color vision some time ago, the relationship between the chemistry and psycho-biology of odors remains unclear up to the present day. Although a series of investigations have demonstrated that this relationship exists, the descriptive and explicative aspects of the proposed models that are currently in use require greater sophistication. One reason for this is that the algorithms of current models do not consistently consider the possibility that multiple chemical rules can describe a single quality despite the fact that this is the case in reality, whereby two very different molecules can evoke a similar odor. Moreover, the available datasets are often large and heterogeneous, thus rendering the generation of multiple rules without any use of a computational approach overly complex. We considered these two issues in the present paper. First, we built a new database containing 1689 odorants characterized by physicochemical properties and olfactory qualities. Second, we developed a computational method based on a subgroup discovery algorithm that discriminated perceptual qualities of smells on the basis of physicochemical properties. Third, we ran a series of experiments on 74 distinct olfactory qualities and showed that the generation and validation of rules linking chemistry to odor perception was possible. Taken together, our findings provide significant new insights into the relationship between stimulus and percept in olfaction. In addition, by automatically extracting new knowledge linking chemistry of odorants and psychology of smells, our results provide a new computational framework of analysis enabling scientists in the field to test original hypotheses using descriptive or predictive modeling.

## Author summary

An important issue in olfaction sciences deals with the question of how a chemical information can be translated into percepts. This is known as the stimulus-percept problem. Here, we set out to better understand this issue by combining knowledge about the chemistry and cognition of smells with computational olfaction. We also assumed that not only one, but several physicochemical models may describe a given olfactory quality. To achieve this aim, a first challenge was to set up a database with ~1700 molecules characterized by chemical features and described by olfactory qualities (e.g. fruity, woody). A second challenge consisted in developing a computational model enabling the discrimination of olfactory qualities based on these chemical features. By meeting these 2 challenges, we provided for several olfactory qualities new chemical models describing why an odorant molecule smells fruity or woody (among others). For most qualities, multiple (rather than a single) chemical models were generated. These findings provide new elements of knowledge about the relationship between odorant chemistry and perception. They also make it possible to envisage concrete applications in the aroma and fragrance field where chemical characterization of smells is an important step in the design of new products.

## Introduction

Around the turn of the century, with its acknowledgement as an object of science by the Nobel society [1] the hidden sense associated with the perception of odorant chemicals, hitherto considered superfluous to cognition, became a focus of study in its own right. Odors are emitted by food, which is a source of pleasure [2]; they also influence our relations with others [3]. The olfactory percept encoded in odorant chemicals contributes to our emotional balance and wellbeing: olfactory impairment jeopardizes this equilibrium [4,5].

Neuroscientific studies have revealed that odor perception is the consequence of a complex phenomenon rooted in the chemical properties of a volatile molecule (described by multiple physicochemical descriptors) further detected by our olfactory receptors in the nasal cavity [6]. A neural signal is then transmitted to central olfactory brain structures [7]. At this stage, a complete neural representation, called “odor” is generated and then, it can be described semantically by various types of perceptual qualities (e.g., musky, fruity, floral, woody etc.). While it is generally agreed that the physicochemical characteristics of odorants affect the olfactory percept, no simple and/or universal rule governing this Structure Odor Relationship (SOR) has yet been identified. Why does one odorant smell of rose and another smell of lemon? Given the fact that the totality of the odorant message was encoded within the chemical structure, chemists have tried for a long time to identify relationships between chemical properties and odors. Topological descriptors, eventually associated with electronic properties or molecular flexibility, have been tentatively connected to odorant descriptors. For instance, molecules carrying a sulfur atom and/or having low molecular weight or low structural complexity are often rated as unpleasant [8–10]. In addition to the hedonic valence of odors, others have looked for predictive models describing odor perception and quality (see [11–14]). Indeed, this was the aim of a crowd-sourced challenge recently proposed by IBM Research and Sage called DREAM Olfaction Prediction Challenge. The challenge resulted in several models that were able to predict pleasantness and intensity as well as 8 out of 19 semantic descriptors (namely “garlic”, “fish”, “sweet”, “fruit”, “burnt”, “spices”, “flower” and “sour”) with an average correlation of predictions across all models above 0.5 [15].

Although these investigations brought evidence that chemical features of odorants can be linked to odor perception, the stimulus-percept problem raised a number of issues. For instance, the stimulus-percept relationship is generally viewed as bijective in that one physicochemical rule describes or predicts one quality. However, some cases suggest the existence of more than a single rule to relate chemistry and perception. Indeed, chemicals belonging to different families can trigger a “camphor” or a musky smell [16]. On the other hand, a single chiral center can render a compound odorless or shift its perceived odor completely, as is the case for (+) and (-)-carvone [17]. These examples strengthen the notion that the connections between the chemical space and the perceptual space are subtler than previously thought with multiple physicochemical rules describing a given quality. At best, the bijective SOR rules may be only be applicable to a very small fraction of the chemical space, with the remaining part of the perceptual space being best described using a multiple rules approach. The complexity of available databases, they include both thousands of chemical properties and a large heterogeneity in perceptual descriptions, [18–21] means that the manual generation of multiple rules is not feasible. In other words, to better understand the stimulus-percept issue in olfaction, there is a clear need to extract knowledge automatically and in an intelligible manner. Such an approach is positioned upstream of predictive modeling since it will enable modeling that extracts descriptive rules from the data that link subgroups belonging to both chemical and perceptual spaces. The main aim of our study was to develop such a computational framework to discover new descriptive structure-odor relationships.

To achieve this, we first set up a large database containing more than 1600 odorant molecules described by both physicochemical properties and olfactory qualities. We then developed an original methodology based on the discovery of physicochemical descriptions distinguishing between a group of objects given a target or class label, namely odor qualities. This approach has been widely studied in Artificial Intelligence (AI), data mining and machine learning. Specifically, supervised descriptive rules were formalized through subgroup discovery, emerging pattern/contrast-sets mining [22]. In all cases, we face a set of objects associated with descriptions and these objects are related to one or several class labels. This new pattern mining method, a variant of redescription mining [23], allows the discovery of pairs consisting of a description (of physicochemical properties) and a label (or sub-set of labels, olfactory qualities). The strength of the rule (SOR in our application) is evaluated through a new quality-control measure detailed in the Methods section.

## Methods

### Olfaction database

We designed and set up a database describing odorant molecules by both their perceptual and physicochemical properties. Here, data from different sources were extracted and grouped: (i) for odorant identification and olfactory qualities, we referred respectively to the PubChem website (https://pubchem.ncbi.nlm.nih.gov/) and the textbook by Arctander [24]; (ii) for physicochemical properties, we referred to the Dragon software package (http://www.talete.mi.it/index.htm).

Olfactory qualities were thus gathered from the book “Perfume and Flavor Chemicals”, published in 1969 by Steffen Arctander. In this book, Arctander gives a complete description, including olfactory and trigeminal qualities as well as flavors, of 3102 odorants (detailed physicochemical properties of 1689 odorants among these 3102 odorants were retrieved, see below). These odorants were further identified by chemical name, molecular weight and corresponding olfactory qualities. Here, the 74 olfactory qualities selected by Chastrette and colleagues [25] were used as a reference list. These qualities were selected in a study of the whole of Arctander’s book by excluding those that did not provide qualitative olfactory information and those that were the least frequent.

Note that before selecting this source, we ran a comparison with other existing Atlases and websites used for research, teaching and applicative purposes: specifically, the Dravnieks Atlas [26], the Boelens Atlas (see [27]), and the Flavornet website (http://www.flavornet.org). These sources (atlases, book and website) were compared along a series of parameters (the comparison took into account all odorants for which we collected CID numbers). The first parameter of interest was the number of molecules studied in the source, and was respectively 1689, 138, 263, and 660 for the Arctander, the Dravnieks, the Boelens and the Flavornet (here, only molecules for which we found a PubChem Compound Identification or CID are taken into account). The second parameter was the number of evaluators (and their expertise level) who smelled the compounds and provided the olfactory qualities: one trained evaluator for the Arctander, a large panel of evaluators for the Dravnieks (although there seems to be a large heterogeneity in the expert profile of these panelists, and little information as to the extent of training that panelists were given), six trained evaluators for the Boelens, and no information is given regarding the panelists for the Flavornet website. Third, when considering the way olfactory qualities were collected in the source, both the Arctander and the Flavornet used a binary format (presence/absence of quality), and both the Dravnieks and the Boelens used a scale of intensity or agreement. Fourth, we compared the number of olfactory qualities used in each atlas/book/website and observed the following distribution (the average number of qualities per molecule is in brackets): 74 (2.88) for the Arctander, 146 (29.99) for the Dravnieks, 30 (12.86) for the Boelens, and 197 (2.72) for the Flavornet. Note also that the minimum (and the maximum) number of qualities for one molecule was: Arctander (min: 1; max: 10), Dravnieks (min: 5; max: 52), Boelens (min: 0; max: 22), Flavornet (min: 1; max: 5).

Thus, this analysis showed that whereas some sources are characterized by a large number of molecules (e.g. Arctander and Flavornet), others contain only a limited number of odorants (e.g. Boelens and Dravnieks). Moreover, there is great heterogeneity between these different sources with regards to the number and the degree of expertise of the evaluators. Some sources involve a large number of evaluators but with heterogeneous profiles (e.g. Dravnieks) and others involve a limited number of experts (e.g. Boelens and Arctander). Finally, whereas some sources have, on average, between 10 and 30 qualities per odorant (e.g. Boelens and Dravnieks), the average number is around three for others (e.g. Arctander and Flavornet). In view of these parameters, and because the descriptive approach used in this study requires a large database, we used the Arctander book because it contained the highest number of odorant molecules (1689) and a reasonable number of qualities per odorant (2.88 on average).

Odorant physicochemical properties were then obtained using Dragon, a software application that enables the calculation of 4885 molecular descriptors (Talete). Descriptors included in our dataset ranged from the simplest atom types, functional groups and fragment counts, to topological and geometrical descriptors. As Dragon requires 3D structure files, these were collected from the PubChem website (https://pubchem.ncbi.nlm.nih.gov) by using the compound identifier number of each odorant (CID). Individual odorant CIDs were obtained by using the CAS Registry Number and/or the chemical name of the odorant as an entry in the PubChem website. In total, 1689 CIDs were found for the 3102 odorants. In the following section, we study the set *M* of odorant molecules that are described by *n* physicochemical properties denoted *F*. Each property *fi* ∈ *F* is a function that associates a real value with a molecule: *fi*: *M* → *image*(*fi*) with image(fi) an interval of R. The olfactory qualities are denoted by *O* and *class* is a mapping that associates a subset of *O* to a molecule: *class*: *M* → 2*O*.

### The developed algorithm

Here, we developed an original subgroup discovery approach to mine descriptive rules that specifically characterize subsets of olfactory qualities (*O*). The specificity of this approach is intended to be able to extract rules with several olfactory qualities as targets, and also to treat unbalanced classes robustly, i.e., the fact that some olfactory qualities are very rare (e.g. “musty”) compared to others (e.g. “fruity”). Subgroup discovery is a generic data mining method aimed at discovering regions in the data that stand out with respect to a given target.

We instantiated this framework in order to identify the conditions on some odorant physicochemical properties that are strongly associated with olfactory qualities.

A *structure odor rule* (*SORule*), denoted *D → Q*, is defined by a physico- chemical description *D* and a set of olfactory qualities *Q ⊆ O*. The description is a set of n intervals D = ⟨[*x1*,*y1*],[*x2*,*y2*],…,[*xn*,*yn*]⟩, each being a restriction on the value image of its corresponding physicochemical property: [*xi*, *yi*] ⊆ *image*(*fi*).

The molecules whose values on physicochemical descriptors belong to the intervals of the description D are members of the coverage of D:

$$coverage(D)\;=\;\{m\;\in M\;\;\forall i\;=\;1\ldots n,\;x_{i}\leq f_{i}(m)\leq y_{i}\}$$

We count the number of molecules in the coverage with *support*(D) = |*coverage*(D)|.

The quality of a rule is evaluated with respect to the olfactory qualities of the molecules in its coverage. First, the *precision* measure gives the proportion of the molecules of the coverage of D that also have (part of) the olfactory qualities *Q*:

$$P(D\to Q)\;=\;\frac{\left|\{m\;\in coverage(D)\;\;class(m)\subseteq Q\}\right|}{support(D)}$$

This is the percentage of times the rule is triggered for molecules whose qualities are in *Q*. On the other hand, it is also important to know if the rule covers all the molecules of quality *Q*. This is what the *recall* measure evaluates:

$$R(D\to Q)\;=\;\frac{\left|\left\{m\;\in coverage(D)\;\;class(m)\;\subseteq Q\right\}\right|}{\left|\left\{m\;\in M\;\;class(m)\;\subseteq Q\right\}\right|}$$

These two measures behave in opposite ways: when one increases, the other decreases. One way to globally evaluate a rule is to use the F_1_ measure, the harmonic mean between the precision and recall measures:

$$F_{1}(D\to Q)\;=\;2\frac{P(D\to Q)R(D\to Q)}{P(D\to Q)+R(D\to Q)}$$

As mentioned above, the olfactory qualities are more or less frequent in the data. To take that into account, the F_β_ measure gives more importance to the precision measure for rare olfactory qualities, while favoring the recall measure for frequent qualities:

$$F_{\beta }(D\to Q)\;=\;\frac{\left(1+\;\beta (support(Q))P(D\to Q)R(D\to Q\right)}{\beta (support(Q))P(D\to Q)+R(D\to Q)}$$

with *support* (*Q*) = |{*m* ∈ *M* |*class*(*m*) ⊆ *Q*}| and

$$\beta (x)\;=\;{\left(0.5\;\times \;(\;1\;+\text{tanh}\;\left(\frac{x_{\beta }-x}{l_{\beta }}\right))\right)}^{2}$$

Here, the terms xBeta and lBeta are determinant in choosing the appropriate sigmoid model, and are values that can be set by the experimenter. Given that, our approach aims to discover rules *D* → *Q* whose support *support*(*D*) is greater than a threshold minSupp and with |*Q*| is lower or equal to a value maxQual. Those parameters make it possible to identify rules that are supported by sufficient odorant molecules, and also that are specific to a small set of olfactory qualities. The maxQual parameter enforces that the right-hand side of the rule contains a limited number of olfactory qualities to be interpretable by the analyst. Similarly, a maxProp parameter allows to limit the number of (physicochemical) conditions in the left-hand side of the rules.

To illustrate the previous definitions, let us consider the toy olfactory dataset given in Table 1. This dataset contains 6 molecules identified by their IDs *M* = {1,2,3,4,5,6}. Each molecule is described by its molecular weight *MW*, its number of atoms *nAt* and its number of carbon atoms *nC*, that is, *F* = {*MW*, *nAt*, *nC*}. Besides, the molecules are also associated with their olfactory qualities among *O* = {*fruity*, *vanillin*, *woody*}. Let us consider the description

D=⟨[128,151],[23,29],[[9,12]⟩

**Table 1:** Description of the developed algorithm: A toy olfactory dataset.

ID	MW	nAT	nC	Quality/Class
1	150	21	11	vanillin, woody
2	128	29	9	fruity
3	136	24	10	fruity, woody
4	152	23	11	woody
5	151	27	12	fruity, vanillin
6	142	27	10	fruity, vanillin

Its coverage is *coverage*(*D*) = {2, 3, 5, 6}. If we consider the odorant quality *Q* = {*vanillin*}, as there is 2 molecules of *coverage*(*D*) with this quality, the precision of the rule is equal to:

$$P(D\to Q)\;=\;\frac{2}{4}$$

As there are 3 molecules in the whole dataset with that quality, the recall of the rule is:

$$R(D\to Q)\;=\;\frac{2}{3}$$

Its *F*_1_ measure is thus equal to:

$$F_{1}(D\to Q)\;=\;2\frac{2}{7}$$

Detailed information regarding the principle of the algorithm are provided as S1 Text.

## Results

### Olfactory dataset: 1689 odorant molecules described by both olfactory qualities and physicochemical properties

**Fig 1:**
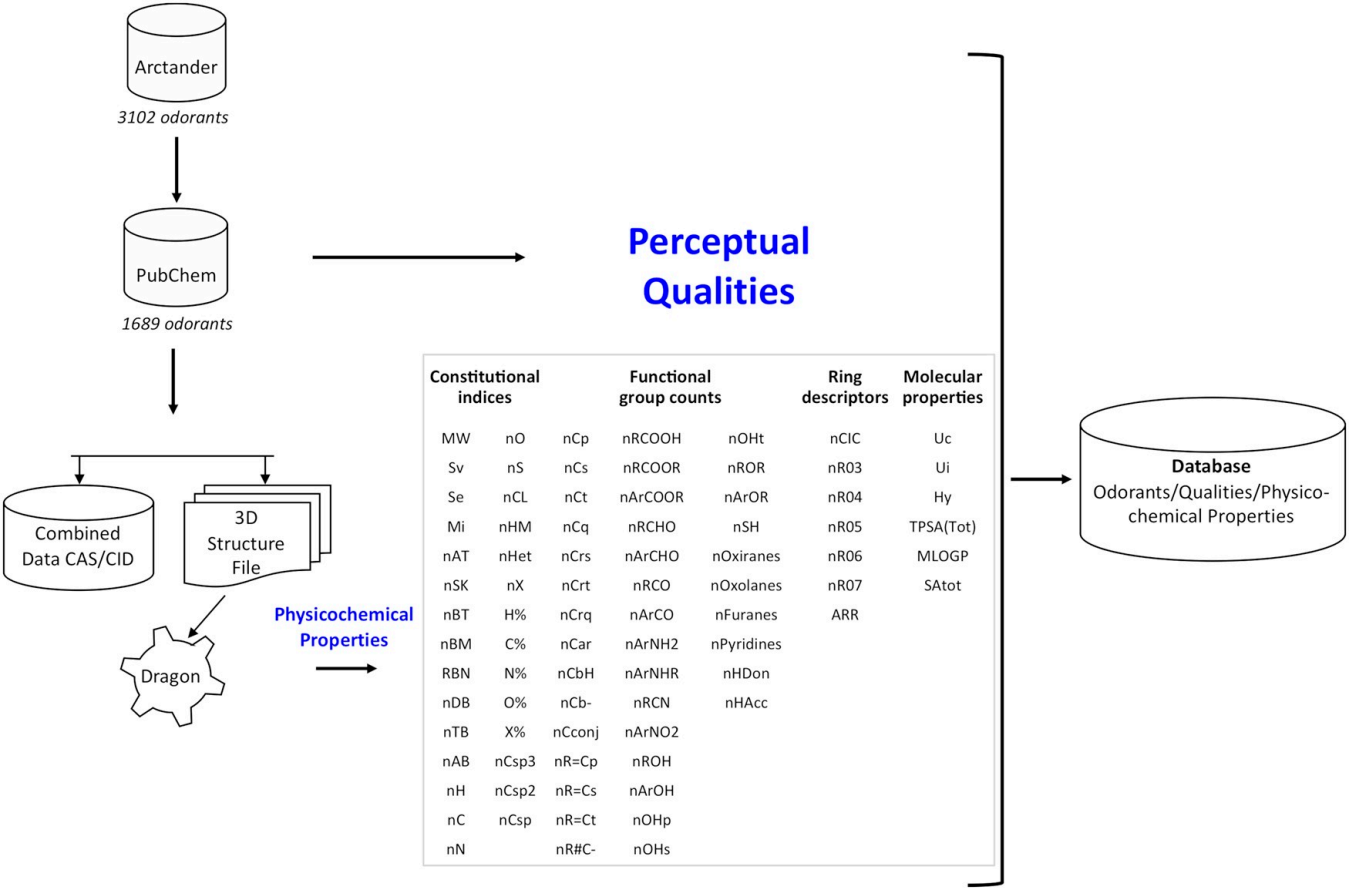
Building process of the database used for the study. It was based on the Arctander’s Book and PubChem databases for determining a total of 74 olfactory qualities. Dragon software was used to obtain 82 physico-chemical properties of the 1689 molecules.

**Fig 2:**
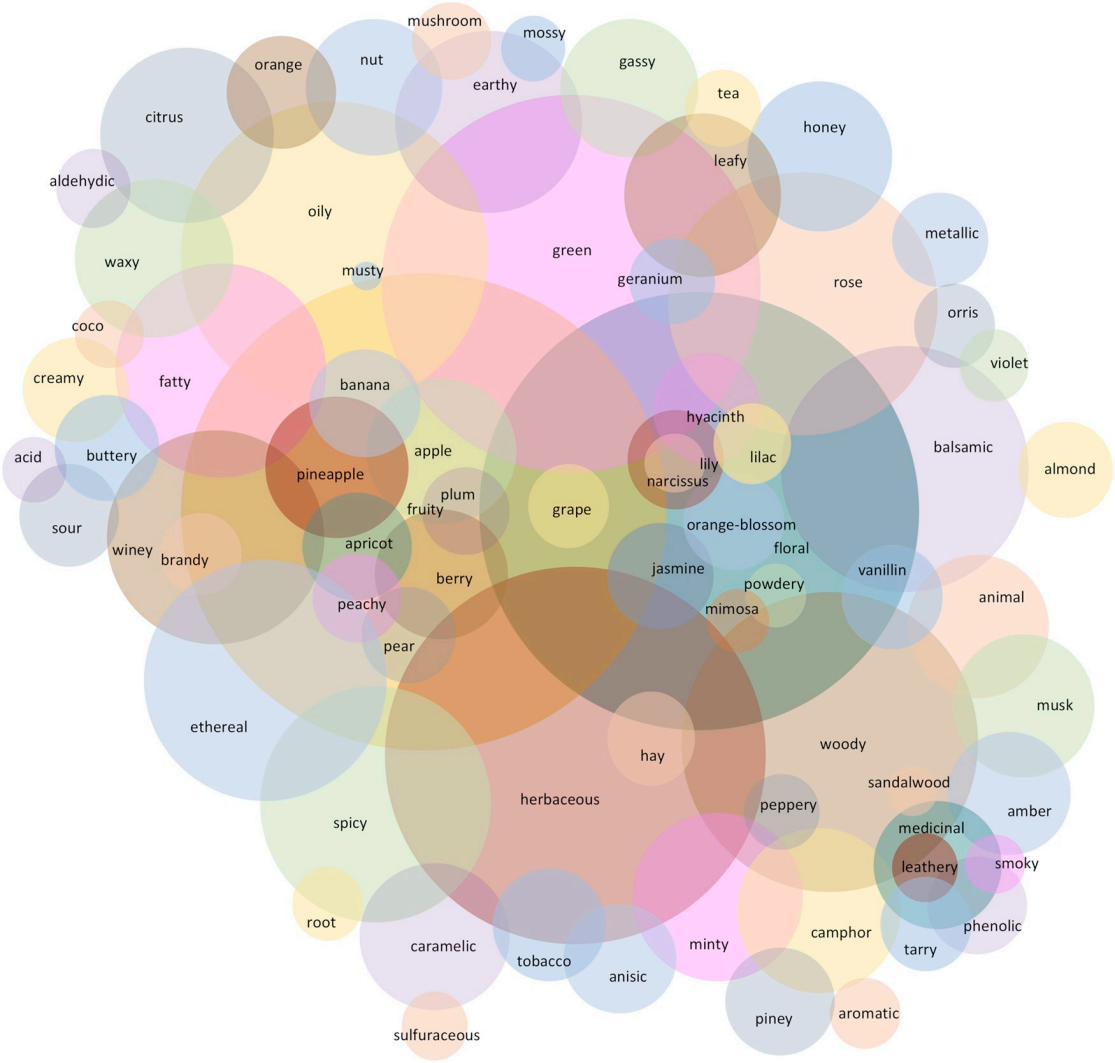
Word-cloud of the 74 studied olfactory qualities.

Our olfactory dataset includes 1689 molecules described by 74 olfactory qualities. The dataset is multi-labeled, each molecule being associated with one or several olfactory qualities. On average, each molecule refers to 2.88 olfactory qualities among the 74 possible labels. Moreover, the frequency of olfactory qualities across odorants is unbalanced: on average a quality is used in 65.79 molecules (standard deviation: 105.28), the maximum is reached for the “fruity” quality (used in 570 molecules), the minimum for musty (used in only 2 molecules). Fig 1 illustrates the entire building process of the database. Fig 2 presents a world cloud of the 74 olfactory qualities.

### Physicochemical properties: Selection and interpretation

With regard to the physicochemical properties, our original database contained more than 4000 physicochemical features. For the purpose of a rational approach where features can be interpreted on a chemical basis, we selected attributes that were relevant, but more importantly easily interpretable. This approach is strongly inspired by the so-called 3D-olfactophore, where such easily interpretable features computed on odorants sharing the same olfactory percept are gathered in the 3 dimensions of space.

Such features are typically Hydrogen bond donor/acceptor, Aromatic cycle, Charged atom, etc. This methodology is typically useful for molecular scientists to learn about structure-property relationships and design new molecules which fulfill the properties of these olfactophores [28]. Here the features we used were a series of physico-chemical properties. Thus, we selected constitutional, topological and chemical descriptors that represent molecular features which can be easily interpreted and extrapolated for further predictive models. They include the following categories: constitutional indices (n = 29; ex. “Molecular weight”), ring descriptors (n = 7; ex. “Number of rings”), functional group counts (n = 40; ex. “Number of esters”), molecular properties (n = 6; ex. “Topological polar surface area”). To select these descriptors, we screened the whole set of descriptors proposed by Dragon. We carefully selected descriptors able to provide information interpretable by any molecular scientist. The cost of selecting interpretable descriptors is a reduction in the description of the dataset. To evaluate the loss of information on the variance of a given molecular dataset, descriptors were computed on a set of 2620 odorants provided by Saito and colleagues [29]. Finally, 347 descriptors remained after filtering the following: correlated (above 0.85), constant for the whole dataset (no variation across parameters), not available for the whole dataset. After the dimensionality reduction, our selected 82 descriptors accounted for 37.2% of the original variance. When choosing randomly 82 descriptors within this set of 347, the variance always falls below 25%, suggesting that our descriptors performed quite well at describing a molecular set with a certain degree of variability. Finally, when projecting the entire set of molecules on to the two first components of a PCA, the dataset remains well split and molecules were still distinguishable.

### Physicochemical descriptive rules: Generation and selection

First, the physicochemical rules were generated for each of the 74 qualities based on the 82 descriptors. This was done using the following parameters: maxoutput (100), beamwidth (30), MaxQual (1), MaxProperties (8), max Supp (700), XBeta (110), IBeta (20), and four different minSupp (5, 10, 20 and 30) (see Methods section and S1 Text for a detailed definition of these parameters). Second, an algorithm search for the best rules or combination of rules (with a maximum of 12 rules) for each of the 74 qualities and the four different minSupp (from 5 to 30). At this stage, the rules or combination of rules were ranked as a function of their Precision. Here, to evaluate the best rule or combination of rules that can describe each quality, we calculated for each rule (or combination of rules) the distance (Euclidian) from the “ideal” situation defined as the data-point with an error of “0” (error was calculated as one minus precision) and the best recall (value of 1 in the y-axis, meaning that all molecules that belong to the quality are described by these physicochemical rules). The point(s) with the smallest distance was (were) selected as the best rule or combination of rules for a given quality.

**Fig 3:**
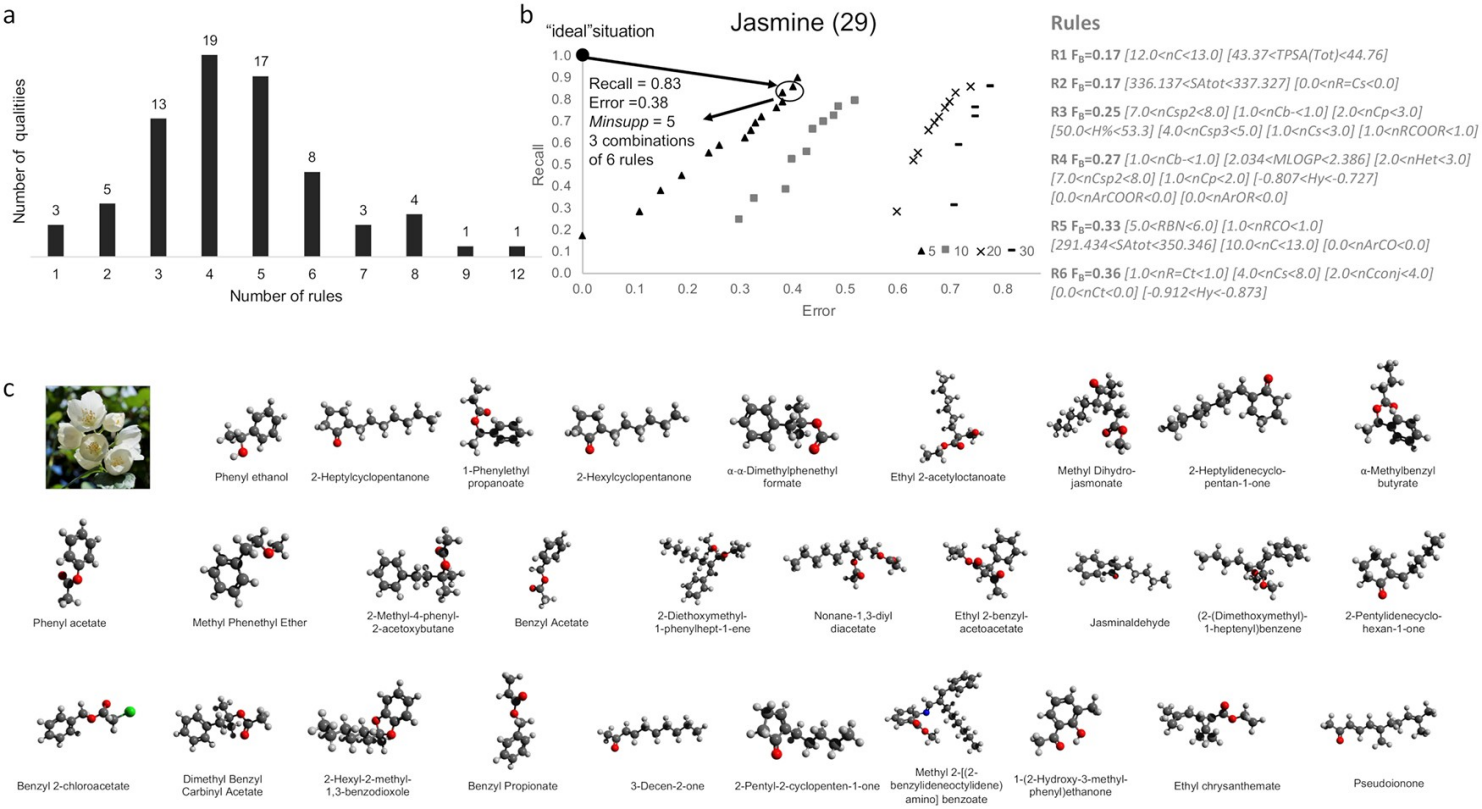
Rules and combinations of rules. (a) Histogram showing the number of rules (x-axis; one to twelve) used to describe the 74 olfactory qualities (number of olfactory qualities, y-axis). (b) Example of the selection of the best rule or combination of rules based on the calculation of Euclidean distance from the “ideal” point (error “0”, recall “1” indicated as ● in the graph) for the olfactory quality jasmine. The number in parenthesis indicates the number of molecules described by this quality. The symbols (▲, ■, x, -) below the graph, indicates the different minSupp used for computation. The right panel indicates the combination of rules selected. (c) Chemical structure and name of the 29 molecules described as “jasmine”.

From this selection, we built a list of rules and/or combination of rules for each quality (see S1 Table). We showed that around 90% of the olfactory qualities were described by 1 to 6 rules and 66% (49 qualities among 74) were described by 3, 4 or 5 rules (see Fig 3a). Moreover, for the same quality, different rules or combinations of rules were selected because their distance to the “ideal” situation (recall: 1; error: 0) was the same (see an example in Fig 3b). Fig 3c shows an example of the chemical structure of the molecules described by the same quality (jasmine here) and rules/combinations of rules.

**Fig 4:**
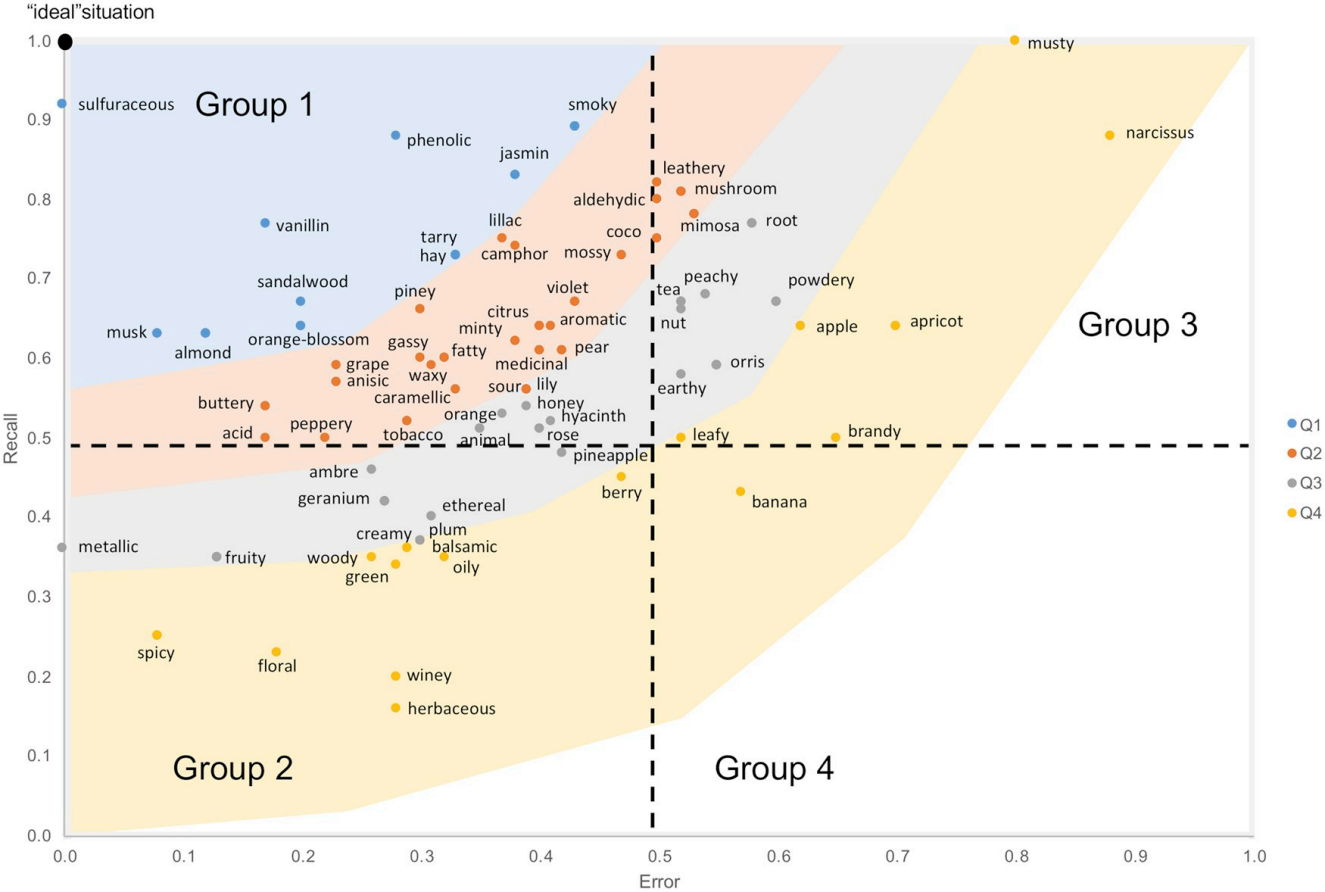
Quartiles and group distribution of the best rules describing the 74 qualities. Quartile distribution was based on the Euclidean distance of a rule/combination of rules from the “ideal” point (error of “0”, recall of “1” indicated as ● in the graph). Quartiles are indicated by different colors (Q1 = blue, Q2 = pink, Q3 = grey, Q4 = orange). Group distribution (indicated as Group 1, 2, 3, and 4) was based on error rate (0.5) and recall (0.5). The 4 groups are separated by the dotted horizontal and vertical lines and indicated in the figure.

To compare olfactory qualities according to their description by physicochemical rules, we plotted all physicochemical rules (and/or combination of rules) of each quality in a 2D space comprising error (x-axis) and recall (y-axis) (Fig 4). As can be seen, whereas some qualities were close to the “ideal” situation others were very far. First, 38 qualities (51.35%, named “Group 1”) exhibited an error rate lower than 0.5 and a recall greater than (or equal to) 0.5 (sulfuraceous, vanillin, phenolic, musk, sandalwood, almond, orange-blossom, jasmine, hay, tarry, smoky, lilac, piney, camphor, grape, anisic, buttery, gassy, fatty, waxy, acid, minty, aromatic, mossy, violet, citrus, peppery, caramelic, medicinal, tobacco, pear, lily, sour, orange, animal, honey, hyacinth, rose). Second, 17 qualities (22.97%, named “Group 2”) exhibited an error rate lower than 0.5 but a recall lower than 0.5 (amber, geranium, metallic, fruity, pineapple, ethereal, plum, woody, balsamic, creamy, green, berry, oily, spicy, floral, winey, herbaceous). Third, 18 qualities (24.32%, named “Group 3”) showed an error rate greater than (or equal to) 0.5 and a recall greater than (or equal to) 0.5 (leathery, aldehydic, mushroom, coco, mimosa, tea, nut, root, peachy, earthy, powdery, orris, apple, leafy, apricot, musty, brandy, narcissus). Fourth, one quality (1.35%, named “Group 4”) showed an error rate greater than (or equal to) 0.5 and a recall lower than 0.5 (banana).

To further examine whether the generated physicochemical rules were specific to a given perceptual quality, in other words whether they provided a good and relevant model, we used Bootstrap confidence intervals to evaluate whether the generated F-measure of the rules/models was significative. Here, knowing that a given set of rules covers X molecules, we sampled 100,000 sets of X molecules (with replacement) and calculated the F-measure of each sample according to the studied quality. Next, the confidence intervals (CI: 99%) of these sets were computed. Afterwards, the F-measure of the set of discovered rules was compared to this CI. Results showed that for all 74 qualities, the F-measure was significant in that its value was outside (and greater) the CI at 99%.

Finally, to examine how the model built with 82 physicochemical descriptors performed compared to a model built with all 4000 descriptors, we calculated the F-measure for each quality (computed on the basis of all sets of rules) in both types of models. Results showed that, on average, the F-measure was significantly greater (p<0.0001) in the model with 82 physicochemical descriptors (mean = 0.592, SEM = 0.012) compared to the model with all 4000 descriptors (mean = 0.487, SEM = 0.011), reflecting that the use of a small but explicative and intelligible set of descriptors enhances performance.

To sum up, we provide here a computational framework that enables the automatic extraction, from a complex and heterogeneous dataset, descriptive rules linking subgroups in a chemical space onto subgroups in a perceptual space. As can be seen in Fig 3a, only 3 qualities could be best described by a single physicochemical rule whereas more than two thirds of the qualities needed between 3 and 5 rules to be described. When dealing with the confidence of the rules, a gradient was observed whereby some rules were associated with a good rate of recall and minimum rate of error, whereas other rules exhibited a lower confidence in describing olfactory qualities. Note that all the generated rules are available to the reader in S1 Table. The computational approach that we developed is available at the following address: https://projet.liris.cnrs.fr/olfamine/

### Interpretation of the physicochemical rules

Here, we analyzed some of the best-known qualities in the field of olfactory evaluation, namely "fruity", "floral", "woody", "camphor", "earthy", "spicy", "fatty". The analysis of the rules and combinations of rules (see S1 Table), shows that the number of rules is quite high for these qualities ranging from six (floral), seven (camphor, earthy), eight (spicy, woody), nine (fatty) to twelve (fruity). From a physicochemical point of view, translated into interpretable rules, the floral quality is characterized by either aromatic and strongly hydrophobic molecules or non-aromatic and moderately hydrophobic odorants. For camphor, molecules are rather small in size, moderately hydrophobic, and eventually cyclic. The earthy quality is characterized by moderately hydrophobic molecules with unsaturations. The spicy quality is characterized by rather rigid molecules, eventually aromatic. Woody quality includes hydrophobic molecules, rather not cyclic nor aromatic. For the fatty, the molecules have a larger carbon-chain skeleton which is highly hydropobic with aldehyde or acid functions. Finally, for the fruity quality, molecules are described as having moderate hydrophobicity and being medium to large in size.

To push the interpretation further, we examined qualities associated with generated physicochemical rules with the highest level of confidence. Here, we attempted (i) to understand the rules based on a priori knowledge and (ii) to examine whether the rules could raise new scientific assumptions.

We analyzed a total of eleven qualities corresponding to the first quartile of the distribution of all rules. Based on the Euclidian distance to the “ideal” situation; 473 rules were generated by our analysis (see Fig 4). These qualities were: sulfuraceous, vanillin, phenolic, musk, sandalwood, almond, orange-blossom, jasmine, hay, tarry, smoky.

The “sulfuraceous” quality was described as follows: *R1*: *[0*.*0<nCsp2<0*.*0] [0*.*0<nHAcc<0*.*0] [11*.*611<Se<22*.*069] [144*.*039<SAtot<222*.*269] [0*.*0<TPSA(Tot)<50*.*6]; R2*: *[1*.*0<nS<2*.*0] [1*.*0<nC<6*.*0] [0*.*0<N%<0*.*0] [25*.*0<C%<33*.*3] [38*.*8<TPSA(Tot)<64*.*18]; R3*: *[1*.*0<nS<2*.*0] [-0*.*264<Hy<0*.*323] [102*.*715<SAtot<222*.*269] [0*.*0<O%<6*.*3]*. These descriptions suggest, somewhat intuitively, that sulfuraceous odorants encompass molecules with one or two sulfur atoms and are moderately heavy, with a maximum of six carbon atoms.

Four rules defined the “phenolic” quality: *R1*: *[216*.*155<SAtot<218*.*661] [0*.*0<nCrs<0*.*0] [0*.*0<nOHp<0*.*0] [30*.*4<C%<45*.*0] [0*.*0<Ui<2*.*322]; R2*: *[1*.*117<Mi<1*.*118] [-0*.*768<Hy<-0*.*158] [0*.*0<nR = Ct<0*.*0] [43*.*5<H%<50*.*0] [0*.*0<nOxiranes<0*.*0] [0*.*0<nR = Cp<0*.*0]; R3*: *[2*.*807<Uc<2*.*807] [3*.*0<nCp<5*.*0] [0*.*4<ARR<0*.*545] [2*.*0<Ui<2*.*0] [-0*.*888<Hy<-0*.*277] [37*.*8<C%<40*.*0] [0*.*0<nOHt<0*.*0] [0*.*0<nOHp<0*.*0]; R4*: *[0*.*6<ARR<0*.*75] [1*.*0<nArOH<2*.*0] [2*.*807<Uc<3*.*17] [170*.*356<SAtot<222*.*475] [0*.*893<MLOGP<2*.*778] [0*.*0<nArCO<0*.*0]*. Thus, odorants having a “phenolic” quality are of moderate size, with few unsaturations and low hydrophilicity (and high lipophilicity). It can be regarded as a cyclic molecule. A good consistency is observed between the 4 rules.

For “vanillin”, the following rules were observed: *R1*: *[0*.*5<ARR<0*.*545] [3*.*0<nCb-<4*.*0] [3*.*0<nHAcc<3*.*0] [1*.*0<nArOR<2*.*0] [0*.*0<nR = Cp<0*.*0] [0*.*0<nArCO<0*.*0] [38*.*1<C%<46*.*2]; R2*: *[3*.*0<nCb-<3*.*0] [3*.*0<nO<3*.*0] [0*.*0<nArCOOR<0*.*0] [-0*.*727<Hy<0*.*66] [42*.*1<H%<50*.*0] [0*.*0<nArCO<0*.*0] [38*.*1<C%<42*.*3]; R3*: *[2*.*0<nCsp3<2*.*0] [1*.*0<nArOR<2*.*0] [0*.*699<MLOGP<1*.*75] [0*.*0<nArCOOR<0*.*0] [0*.*0<nArCO<0*.*0] [2*.*0<nCb-<4*.*0]*. These descriptions suggest that odorants belonging to this group are mostly cyclic molecule (like the prototypical molecule vanillin), with 3 Hydrogen bond acceptors branched on saturated carbons atoms on an aromatic cycle.

When considering the “musk” quality, the following rules emerged: *R1*: *[3*.*72<MLOGP<4*.*045] [2*.*0<nCrs<15*.*0] [1*.*0<nCIC<1*.*0] [333*.*936<SAtot<436*.*545]; R2*: *[4*.*0<nCb-<6*.*0] [33*.*0<nBT<47*.*0] [0*.*0<nCbH<2*.*0]; R3*: *[0*.*0<RBN<0*.*0] [11*.*0<nCs<16*.*0]; R4*: *[238*.*46<MW<270*.*41] [57*.*1<H%<63*.*8] [402*.*5<SAtot<440*.*301] [0*.*0<nR07<0*.*0] [-0*.*931<Hy<-0*.*763] [0*.*0<ARR<0*.*316] [0*.*0<RBN<12*.*0] [0*.*0<nCt<3*.*0]*. Musky molecules are heavy and hydrophobic compounds. This is reflected by a rather large logP, surface area or molecular weight. From a general point of view, these descriptors reflect well the features of musky odorants.

For the “sandalwood” quality, two rules were observed: *R1*: *[3*.*0<nCrt<5*.*0] [1*.*0<nHDon<1*.*0] [0*.*0<nR04<0*.*0] [1*.*0<nCrq<2*.*0]; R2*: *[3*.*0<nCrt<5*.*0] [1*.*0<nHDon<1*.*0] [-0*.*429<Hy<-0*.*325] [2*.*0<nR05<3*.*0]*. Sandalwood odorants are quite diverse and minor modifications within their structure can abolish the sandalwood note. The rules which are mined here correspond to models which are very simple and hardly capture the subtlety of this odorant family [28]. The description presented here corresponds to the prototypic beta-santalol structure which has a campholenic skeleton.

The “almond” quality was described by four rules: *R1*: *[0*.*0<nCp<0*.*0] [152*.*443<SAtot<165*.*41] [1*.*0<nO<2*.*0] [2*.*0<Ui<2*.*585]; R2*: *[0*.*706<ARR<0*.*8] [0*.*0<nArCO<0*.*0] [1*.*0<nO<1*.*0] [3*.*0<Uc<3*.*807] [0*.*143<MLOGP<3*.*571] [-0*.*917<Hy<-0*.*71] [0*.*0<nCb-<2*.*0]; R3*: *[1*.*0<nH<5*.*0] [0*.*0<nOxiranes<0*.*0] [1*.*0<nHAcc<3*.*0] [1*.*0<nN<2*.*0] [23*.*79<TPSA(Tot)<90*.*27] [0*.*0<O%<14*.*3] [0*.*0<ARR<0*.*75]; R4*: *[1*.*0<nArCHO<1*.*0] [11*.*0<nBT<20*.*0] [45*.*0<C%<47*.*1] [-0*.*864<Hy<-0*.*668] [1*.*0<nHAcc<2*.*0]*. These descriptions suggest that odorants evoking an almond-like quality are compounds bearing at least one oxygen and/or other hydrogen bond-accepting atom but also bearing an aromatic cycle. This means that the structure bears several unsaturations. These chemicals are thus relatively small and can be compared to the prototypical structure of benzaldehyde.

Four physicochemical rules described the “orange-blossom” quality: *R1*: *[10*.*0<nCsp2<10*.*0] [9*.*23<TPSA(Tot)<58*.*89]; R2*: *[1*.*0<nArNH2<1*.*0] [213*.*361<SAtot<326*.*286] [0*.*0<nR = Cs<0*.*0] [0*.*0<nCt<0*.*0] [37*.*9<C%<51*.*5]; R3*: *[0*.*773<ARR<0*.*857] [39*.*4<H%<45*.*5] [9*.*23<TPSA(Tot)<52*.*32] [3*.*0<nCb-<5*.*0]; R4*: *[47*.*243<Se<53*.*454] [4*.*0<nCbH<9*.*0] [3*.*287<MLOGP<5*.*007] [3*.*0<nHAcc<4*.*0] [0*.*231<ARR<0*.*462]*. These descriptions characterize very diverse structures ranging from very small to medium or large compounds. As a general rule, one can note the presence of unsaturations, consistent with a terpenic structure, associated with a quite hydrophobic feature.

The “jasmine” quality was described by six rules: *R1*: *[12*.*0<nC<13*.*0] [43*.*37<TPSA(Tot)<44*.*76]; R2*: *[336*.*137<SAtot<337*.*327] [0*.*0<nR = Cs<0*.*0]; R3*: *[7*.*0<nCsp2<8*.*0] [1*.*0<nCb-<1*.*0] [2*.*0<nCp<3*.*0] [50*.*0<H%<53*.*3] [4*.*0<nCsp3<5*.*0] [1*.*0<nCs<3*.*0] [1*.*0<nRCOOR<1*.*0]; R4*: *[1*.*0<nCb-<1*.*0] [2*.*034<MLOGP<2*.*386] [2*.*0<nHet<3*.*0] [7*.*0<nCsp2<8*.*0] [1*.*0<nCp<2*.*0] [-0*.*807<Hy<-0*.*727] [0*.*0<nArCOOR<0*.*0] [0*.*0<nArOR<0*.*0]; R5*: *[5*.*0<RBN<6*.*0] [1*.*0<nRCO<1*.*0] [291*.*434<SAtot<350*.*346] [10*.*0<nC<13*.*0] [0*.*0<nArCO<0*.*0]; R6*: *[1*.*0<nR = Ct<1*.*0] [4*.*0<nCs<8*.*0] [2*.*0<nCconj<4*.*0] [0*.*0<nCt<0*.*0] [-0*.*912<Hy<-0*.*873]*. This rule characterizes (i) molecules composed mainly of carbons and oxygen atoms, (ii) molecules with an aromatic core and embranchments conferring a large flexibility, and (iii) compounds with an optimal chain length around five carbon atoms. These rules are in line with the prototypical molecule jasmonate.

For the “hay” quality, six rules were generated: *R1*: *[1*.*0<nArCO<1*.*0] [-0*.*164<Hy<0*.*647] [1*.*239<MLOGP<2*.*001]; R2*: *[13*.*3<O%<13*.*6] [2*.*322<Ui<2*.*322] [1*.*191<MLOGP<1*.*75] [0*.*0<nR = Cp<0*.*0] [179*.*198<SAtot<300*.*766]; R3*: *[0*.*556<ARR<0*.*6] [177*.*465<SAtot<205*.*275] [26*.*3<TPSA(Tot)<50*.*44] [-0*.*603<MLOGP<2*.*001] [11*.*1<O%<20*.*0] [0*.*0<nCs<0*.*0] [0*.*0<nPyridines<0*.*0] [1*.*0<RBN<2*.*0]; R4*: *[2*.*0<nCb-<2*.*0] [179*.*249<SAtot<209*.*869] [2*.*322<Ui<2*.*585] [26*.*3<TPSA(Tot)<37*.*3]; R5*: *[1*.*111<Mi<1*.*116] [2*.*0<nHAcc<2*.*0] [2*.*0<nCb-<4*.*0] [0*.*0<nR05<0*.*0] [0*.*0<nCconj<1*.*0] [1*.*49<MLOGP<3*.*719]; R6*: *[0*.*0<RBN<0*.*0] [2*.*0<nO<2*.*0] [0*.*0<nR = Ct<1*.*0] [-0*.*807<Hy<-0*.*668] [26*.*3<TPSA(Tot)<30*.*21] [130*.*383<SAtot<214*.*985] [-0*.*145<MLOGP<2*.*265]*. These rules characterize relatively hydrophobic molecules composed of aromatic cycles, being either heterocyclic or linked to a heteroatom outside of the cycle. These atoms confer to the molecule the possibility to accept Hydrogen bonds.

“Tarry” quality was also described by six rules: *R1*: *[6*.*0<nCsp2<6*.*0] [-0*.*213<Hy<0*.*031] [1*.*348<MLOGP<1*.*859] [0*.*0<RBN<1*.*0]; R2*: *[3*.*0<nCb-<4*.*0] [2*.*807<Uc<2*.*807] [1*.*0<nHet<2*.*0] [18*.*46<TPSA(Tot)<40*.*46] [138*.*18<MW<178*.*3] [38*.*7<C%<40*.*0] [0*.*0<nCs<0*.*0]; R3*: *[0*.*733<ARR<0*.*773] [-0*.*905<Hy<-0*.*158] [1*.*0<nHet<1*.*0] [174*.*318<SAtot<277*.*868]; R4*: *[0*.*0<nDB<0*.*0] [174*.*318<SAtot<175*.*125] [2*.*807<Uc<3*.*585] [15*.*862<Se<17*.*534]; R5*: *[5*.*9<N%<7*.*7] [0*.*6<ARR<1*.*0] [0*.*565<MLOGP<1*.*834] [-0*.*828<Hy<0*.*031] [12*.*753<Se<16*.*636]; R6*: *[-0*.*213<Hy<-0*.*158] [16*.*0<nBT<21*.*0] [0*.*0<nRCOOH<0*.*0] [0*.*0<nROH<0*.*0] [1*.*58<MLOGP<2*.*193] [15*.*79<TPSA(Tot)<29*.*46]*. With regard to this quality, it is not easy to establish specific characteristics of the molecules of this group, but overall these molecules are flexible, presenting heteroatoms while having low hydrophilicity due to the presence of double bonds.

Finally, the “smoky” quality is described as follows: *R1*: *[1*.*0<nArOH<1*.*0] [1*.*859<MLOGP<2*.*193] [1*.*117<Mi<1*.*121] [0*.*0<nCconj<0*.*0]; R2*: *[7*.*0<nC<7*.*0] [2*.*807<Uc<2*.*807] [41*.*2<C%<43*.*8] [-0*.*158<Hy<-0*.*107]; R3*: *[0*.*0<RBN<0*.*0] [4*.*0<nCar<5*.*0] [99*.*023<SAtot<129*.*741]*. In this case, a robust rule is hard to establish because the physicochemical descriptors refer either to aromatic compounds with a hydroxyl group or flexible molecules with rotatable bonds.

### Validation of the physicochemical rules in novel odorants

To evaluate the validity of the generated physicochemical rules, we applied them to novel sets of odorants. For a given quality, we checked whether novel odorants that fulfill physicochemical criteria according to our descriptive model indeed evoked significantly more of the studied quality than novel odorants than do not fulfill these physicochemical rules.

To this end, we isolated from 4 different databases, 4 sets of odorants not present in the Arctander database and therefore not used to build the descriptive rules. These databases were from the Dravnieks study [26] (n = 45; i.e. 45 odorants not present in our original dataset could be used), the Boelens & Harding study [30] (n = 56), one set from the Keller et al. study [15] (n = 118), and one set from the Licon et al. study [31] (n = 19). Within each of these four novel sets, olfactory quality was coded using a continuous variable (Dravnieks: from 0 to 100; Boelens & Haring: from 0 to 9; Keller et al.: from 0 to 100; Licon et al.: from 0 to 100). Note that, for the Keller et al. study, perceptual data were provided for 2 levels of odorant concentrations (« High » and « Low »).

Our descriptive model was tested in qualities that were common between the Arctander database and these four different databases. Moreover, for statistical purposes and for a given quality, only when the rules were filled for at least five odorants, comparisons were performed between odorants that filled the criteria for the rules and those that did not filled the rules. The qualities that satisfy these criteria were: 1/ for the Dravnieks study: Woody (n = 5), Camphor (n = 5), Earthy (n = 5), 2/ for the Boelens & Haring study: Woody (n = 10), Fruity (n = 9), Green (n = 8) and Balsamic (n = 5), 3/ for the Keller et al. study: Fruity (n = 15), and Sulfuraceous (n = 16; which was compared to a semantically proximal perceptual quality present in the Keller database, namely « Decayed »), and 4/ for the Licon et al. study: Camphor (n = 5).

**Fig 5:**
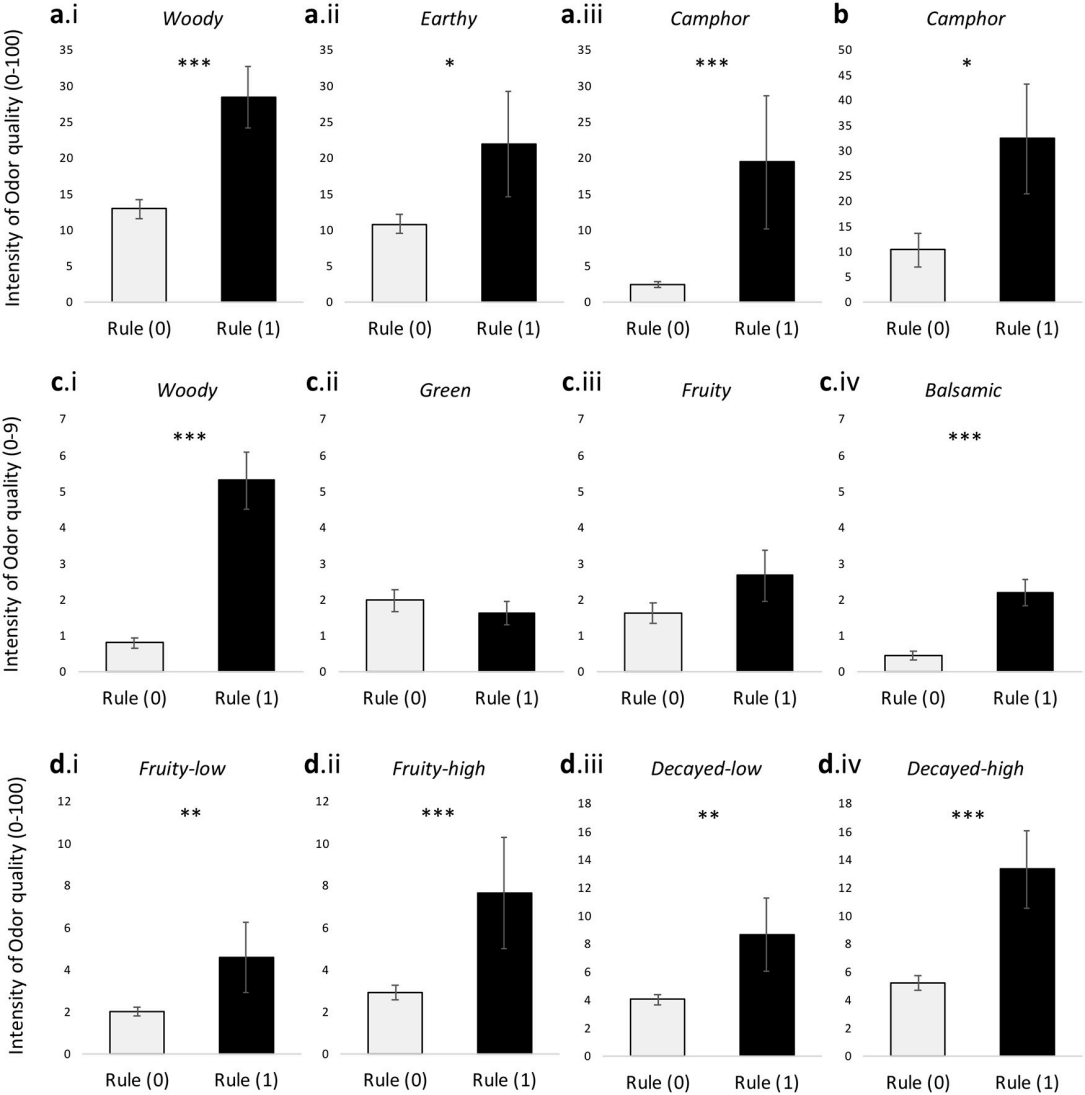
Validation studies in novel odorants from different available databases. Bars correspond to means and error bars to the standard error to the mean (SEM). (a) Dravnieks dataset: odorants that fulfill the criteria for the rules woody (a.i), earthy (a.ii), and camphor (a.iii) from our model (Rule (1), black bars) were significantly more described by these respective qualities than odorants that did not fulfill these physicochemical rules (Rule (0), grey bars). (b) Licon et al. dataset: significant validation was observed for camphor. (c) Boelens & Haring dataset: validation of our rules was observed for woody (c.i) and balsamic (c.iv). (d) Keller et al. dataset: odorants that fulfill the criteria for rules associated with fruity were indeed perceived as more fruity for both concentrations of odorants (d.i: low concentration; d.ii: high concentration). Validation of the rules associated with the quality sulfuraceous was also observed (odorants that fulfill the criteria were perceived as more decayed at both low, d.iii, and high, d.iv, concentrations). ***p<0.001, **p<0.01, *p<0.05.

Results are presented in Fig 5. Within each set, an analysis of variance (ANOVA) comparing perceptual values for a given quality for odorants that fulfill the physicochemical rules (Rule (1), black bars) vs. those that did not fulfill the rules (Rule (0), grey bars) was performed. For the Dravnieks dataset, the statistical analysis revealed that odorants that fulfill the rules for woody, earthy and camphor, were respectively perceived as significantly more woody (F(1,43) = 14.19, p<0.001, η^2^ = 0.248; Fig 5a.i), earthy (F(1,43) = 6.128, p = 0.017, η^2^ = 0.125; Fig 5a.ii) and camphoreous (F(1,43) = 28.63, p<0.001, η^2^ = 0.400; Fig 5a.iii). In the same line, a significant increase in camphor quality was observed for odorants that fulfill the rules for this quality in the Licon et al. dataset (F(1,17) = 6.804, p = 0.018, η^2^ = 0.286; Fig 5b). Validation was also observed within the Boelens & Haring dataset, but the results were more mixed. Whereas a significant increase was observed for woody (F(1,54) = 88.47, p<0.001, η^2^ = 0.621; Fig 5c.i) and balsamic (F(1,54) = 15.86, p<0.001, η^2^ = 0.227; Fig 5c.iv) in odorants that fulfill the physicochemical rules for these respective qualities, this was not the case for the green quality (F(1,54) = 0.227, p = 0.636, η^2^ = 0.004; Fig 5c.ii). On a descriptive level, Fig 5c.iii shows that odorants that fulfill the physicochemical criteria for the quality fruity seem to be perceived as more fruity, but this was not significant (F(1,54) = 1.989, p = 0.164, η^2^ = 0.036). However, when considering the Keller et al. dataset, validation was reached for fruity: odorants that fulfill criteria for the fruity quality were perceived as more fruity (for both low (F(1,116) = 9.219, p = 0.003, η^2^ = 0.074; Fig 5d.i) and with high levels of concentrations (F(1,116) = 11.76, p<0.001, η^2^ = 0.092; Fig 5d.ii)), than odorants that did not fulfill the rules. The statistical analysis of this dataset shows also that odorants that fulfill the physicochemical criteria for the quality sulfuraceous were perceived as more decayed at both low (F(1,116) = 10.49, p = 0.002, η^2^ = 0.083; Fig 5d.iii) and high levels of concentrations (F(1,116) = 24.42, p<0.001, η^2^ = 0.174; Fig 5d.iv).

To sum up, the present validation involved four sets of stimuli for a total of 238 odorants. It allowed us to test the descriptive model on seven perceptual qualities and for six of them (woody, earthy, camphor, balsamic, sulfuraceous, fruity), the rules generated by our model have been consistent with the ratings provided in these independent datasets.

## Discussion

The interaction between the odorant molecule and the olfactory receptor(s) induces a percept called “odor”. Chemists have previously attempted to characterize this phenomenon by working to obtain descriptive and/or predictive rules connecting physicochemical properties to odors [32]. Such is the case with olfactophores or the exploitation of more specific molecular features for predicting intensity or pleasantness [8,11,33,34]. Recently, a large database of compounds as well as a large number of human panelists were used in order to predict percepts, intensity and pleasantness [15]. In our study, we also considered that, to a certain extent, the odor quality of a molecule is encoded in its chemical structure. Our aim was to provide a descriptive model of the relationship between molecules and their perceived odors. To achieve this aim, we set up a new computational framework that considers the scientific assumption that, rather than relying on single physicochemical descriptions, the relationship between the chemical space of odorants and the perceptual space of odors should be examined through multiple descriptions. We developed a new method based on a subgroup discovery algorithm to mine descriptive (physicochemical) rules characterizing specific subsets of class labels (olfactory qualities). Thanks to this data-mining approach, we were able to provide new descriptive structure-odor rules with a gradient of confidence (taking into account both the recall and the precision) that varied from one quality to another. Validation of these descriptive models was achieved for a series of olfactory qualities associated with rules with medium levels of confidence (woody, earthy, balsamic, fruity) to higher levels of confidence (sulfuraceous and to a less degree camphor).

Our findings contribute to a better understanding of the olfactory system by elucidating the relationships between the chemistry and the psychobiology of smells. Indeed, the function of the olfactory system is to detect and discriminate volatile environmental molecules in order to make sense of them. This implies the construction of dedicated percepts that can influence behavior. In order to understand this system, relating the worlds of chemistry and perception is a requirement. Our findings provide descriptive elements of responses and highlight the physicochemical rules that describe olfactory perceptual qualities. Beyond these aspects, our algorithm would benefit from a more systemic approach through the inclusion of neurobiological representational states, ranging from olfactory receptors and olfactory bulb to primary and secondary olfactory areas. This will allow us to better understand how the interaction between the chemical features of odorants and olfactory receptors is mediated and processed in the brain to build olfactory percepts.

One question that may be raised from the current finding is how our descriptive approach is different from other machine learning methods and how it may help chemists and neuroscientists interested in olfaction solve scientific issues? In contrast to classical predictive machine learning tasks where the goal is to turn the data into an as-accurate-as-possible prediction machine, exploratory data analysis such as ours aims to automatically discover new insights about the domain in which the data was measured (e.g., olfaction). To this end, the notion of interpretability is fundamental as it is the premise of descriptive rules. Indeed, these rules are composed of conjunctions of conditions on attributes that conclude on some olfactory qualities. In contrast to black-box models, these rules, assessed by intuitive and mathematically well-funded measures are easy to assimilate for a domain expert. This, in turn, makes development of new hypotheses possible. In sum, our data-mining method should be regarded as an approach that can extract knowledge from a dataset characterized by its complexity, size and heterogeneity. Our approach is therefore situated at the upstream of any hypothetical-deductive approaches. The generation of descriptive rules allows researchers to start such a hypothetical-deductive approach, and to formulate new scientific assumptions, to establish an experimental methodology and finally to develop and test the validity of predictive models.

Our algorithm has made it possible to extract significant knowledge about a series of olfactory qualities. First, qualities with a chemical terminology (sulfuraceous, vanillin, phenolic) have a great reliability in the rules generated. These rules contained expected attributes such as the presence of sulfur atoms to describe “sulfuraceous odors”, suggesting that our algorithm was efficient in extracting relevant and meaningful knowledge. Our results went beyond the sole description of these expected physico-chemical attributes. The generated rules contained also unexpected features such as “phenolic odors”, where the presence of moderate size molecules, with few unsaturations and low hydrophilicity were put forward. Structure-odor relationships for some qualities such as musky [35,36], sandalwood [37–39] and to a lesser degree almond and jasmine [40] have already been explored in the past. Our descriptive model could bring new information for most of these qualities, thus enabling the testing of innovative hypothesis in the field. Importantly, we revealed the existence of descriptive rules for qualities that have not, to the best of our knowledge, been investigated before. These qualities include orange-blossom, hay, tarry and smoky. The generated rules will help scientists to better understand the chemical composition of the stimuli that evoke these odors and bring new insights about the way these molecules can interact with the olfactory system at the receptor level. Last but not least, our approach showed also that it was difficult to generate reliable rules for some qualities, particularly the most represented in the database (e.g., fruity, floral and woody). Although the recall associated with these rules was not high, they were characterized by a low rate of error, and validation was achieved for some of them including the well-known fruity and woody qualities. Finally, it is noticeable that a series of interesting qualities were described by rules with a good level of confidence but may be not precise enough to warrant detailed interpretation at this stage. These qualities are those that belong to the second quartile (Fig 4) and include, for instance, camphor for which validation with novel odorants was performed using two different external datasets.

A methodological issue that may be raised from our study relates to the choice of Arctander’s book in our methodology. Before answering this question, one must detail why such linguistic sources are used in olfactory research. In general terms, whereas emotional reactions are very prominent in olfaction [41], lexical and linguistic processes are relatively limited: spontaneous odor identification performances are around 50% (see [42]). Such an absence has led scientists and those in the industry to develop different sources (atlases, books, websites) listing the olfactory qualities of a series of odorant molecules (Arctander book [24], the Dravnieks Atlas [26], the Boelens Atlas [27], and the Flavornet website (http://www.flavornet.org)). A comparison of these sources led us to consider the Arctander’s book since it contained the highest number of odorant molecules and a reasonable number of qualities per odorant. The book, in being developed by a single scientist, gave the advantage of allowing us to integrate more homogeneous data with less variable response profiles than those collected in other atlases. However, this same feature also opens up the possibility that certain odorants that evoke a given quality could be missed. One should therefore not discard the possibility that certain molecules that evoke, for instance, the quality “fruity” were not considered by our model in the validation phase because they were just below the perceptual threshold set by Arctander for that particular quality. Given the variability of olfactory perception between individuals, it is conceivable that the same quality of “fruity” could have been the perceptual threshold of another rater. As a consequence of these factors, we face a double challenge: on one hand, there is a clear need to implement some flexibility in olfactory databases, whereby a given molecule can be described by one or several qualities with an associated level of confidence instead of a binary response. On the other hand, in order to account for interindividual variability in olfactory perception, olfactory databases need to consist of data from a large number of individuals. Future work will need to overcome these factors, for example, by asking raters to provide a level of confidence alongside each response, or by using a fuzzy logic algorithm in order to provide the model with responses ranging in quality from not at all plausible to extremely plausible. In this way, our model will benefit from a better characterization of olfactory percepts, as the rules generated would be more suited to the complexity of human perception. On a more general front, one interesting perspective in this research field would be to implement a new Atlas that integrates response diversity accompanied by all the strengths present in each individual atlas (see Methods section; large number of molecules, large panel of evaluators in the qualitative description of each odor). Such an atlas could serve as a basis for a large number of: (i) fundamental research studies (to better understand the perceptual olfactory space and its relation to the chemical space and the neuronal space), (ii) applied research studies (to better understand the olfactory properties of new compounds developed by the perfume and flavor industry), (iii) education and teaching actions (to standardize olfactory learning procedures in perfume schools or culinary arts schools).

To sum up, current psychological and biological models of olfaction consider that olfactory perception is not totally universal. Although the sense of smell includes invariant aspects, a wide range of olfactory responses are characterized by their diversity from one person to another. In other words, while some molecules can induce very similar behavioral responses and perceptions among individuals, other molecules induce diverse perceptions, not only between individuals but also within the same person according to physiological and cognitive factors. It is undoubtedly in the invariant part of olfaction that we can establish the best predictive models linking chemistry to perception. In this case, a model including bijective rules can even be considered. Nevertheless, the more one moves towards the area of perceptual space of odors that is characterized by its heterogeneity between individuals, the higher the predictability threshold (i.e. bad prediction) becomes. This variability characterizes what could be called "the glass ceiling of olfactory diversity". New methods are thus needed to break or circumvent this glass ceiling. Such methodology should integrate the notion of multiple rules for linking the chemical space to these diverse perceptions. Our approach is providing some new elements to this challenging issue.

In conclusion, the present findings provide two important contributions to the fields of computation and neurosciences. First, although direct SOR seems illusory for some olfactory qualities if additional protagonists of the sense of smell are not taken into account, our approach suggests that descriptive rules exist for some qualities. Second, the present approach showed that several sub-rules should be taken into account when describing structure-odor relationships. From these findings, by correlating the multiple molecular properties of odors to their perceptual qualities and evoked-neural activities, experts in neuroscience and chemistry may generate new and innovative hypotheses in the field. In terms of application, this work can add to our knowledge of the complex phenomenon of smells and tastes. Indeed, by implementing such a descriptive structure/odor model within a dedicated data-analytics platform we could improve our understanding of the effects of molecular structure on the perception of those objects with highly-valued odorant properties such as foods, desserts, perfumes and flavors. This, in turn, would enable the optimization of product formulation with respect to the needs and expectations of consumers.

## Supporting information

S1 Text Information about the algorithms developed for the discovery of structure odor rules. (DOCX)

S1 Table List of rules and/or combination of rules for each olfactory quality. (XLSX)
