# Supplementary material for: Chemical features mining provides new descriptive structure-odor relationships
Source: PLoS Comput Biol. 2019 Apr 25;15(4):e1006945. doi: 10.1371/journal.pcbi.1006945 (PMC6504111; doi:10.1371/journal.pcbi.1006945)
Supplement: S1 Text — (DOCX) [file pcbi.1006945.s001.docx]

**Supporting Information**

***Algorithms for the discovery of Structure Odor Rules***

Each rule is provided with the aforementioned metrics, and we choose here to extract the set $S$ of the $k$ best rules maximizing the $F_{\beta}$ score. Thus, we search for rules that foster precision for overexpressed olfactory qualities sets, and balance between recall and precision for underrepresented qualities subsets.

*Algorithm principle.* The number of all SORules is exponential. It is given by $\Pi_{i=1}^{n}\frac{{image(f}_{i})\times\left( {image(f}_{i})+1 \right)}{2}\times2^{|O|}$: The first term represents the number of all possible descriptions [1], the second term counts the numbers of olfactory qualities subsets. Consequently, the naive approach, which consists of generating and testing all rules to finally retain only the best, is impossible. The algorithm that we propose for rule discovery makes use of the lattice structure of the rule search space and explores it from the most frequent to the less frequent rules: Rules with highest support are explored first. Beam search, a well-known heuristic in rule discovery, is applied to make the search tractable [2].

The method to extract the set $S$ of the top-$k$ best SORules employs a level wise exploration of the search space of the SORules as illustrated in **Supp Figure 1a**. The pseudo code of the algorithm is given in **Supp Figure 1b** and explained in what follows.

Level 0 contains the most general SORule $R_{0}$:

$\left\langle\begin{aligned} \left[ \min\left( image\left( f_{1} \right) \right)\leq f_{1}\leq\max\left( image\left( f_{1} \right) \right) \right],\ldots, \\ \left[ \min\left( image\left( f_{n} \right) \right)\leq f_{n}\leq\max\left( image\left( f_{n} \right) \right) \right] \end{aligned} \right\rangle\to\emptyset$.

Level 1 is generated by the SORules for which an olfactory quality is added to the set of qualities from the SORule $R_{0}$.

From each SORule of level 1, a set $C$ of specialized SORules is built. The specialized SORules are obtained by either selecting the best interval for a feature (not restricted yet) that maximizes locally the $F_{\beta}$ score or by adding a new olfactory quality to the set of olfactory qualities of the SORule. The computation of the best interval for a feature is detailed in the next paragraph. The set of specialized SORules $C$ is filtered out to remove the SORules $d\to q$ for which the metric is lower than the metric of their generalized SORule (see line 9 of the pseudo code in **Supp Figure 1b**), infrequent SORules (we are interested in large enough SORules because small supports are not significant, see line 10), too large descriptions $d$ (each SORule should be easily interpreted, thus the description should not be too long, see line 11) and too large qualities $q$ (it is not significant to extract SORules for which there are too many olfactory qualities, see line 12). We then filter this set $C$ to only retain the non-similar SORules (line 13). It is indeed a well-known problem in supervised rule discovery that the best rules are highly redundant [3]: for example, the two best rules according to the $F_{\beta}$ score will probably slightly differ having almost the intervals, quality measure and coverage. This filter is described hereafter.

To generated rules of level $i+1$, beam search only retains a fixed number (*beamwidth*) of SORules from the filtered set $C$ at level $i$. The number of SORules is called the beam width (denoted as the $beamWidth$ parameter in the pseudocode). Thus, only the best $beamWidth$ SORules from $C$ w.r.t. the $F_{\beta}$ score are retaining in the beam. This process of specializing the SORules in the beam is recursively performed until the beam becomes empty, and then the result $S$ is filtered (line 19) and only the top-$k$ SORules from $S$ are returned to the user. The illustration of the exploration in the search space is given in **Supp Figure 1c**.

*Finding the best interval.* To specialize a SORule $d\to q$ w.r.t. a feature $f_{l}\in F$, it is required to find the best interval $[x\leq f_{l}\leq y]$ that maximizes the metric $F_{\beta}$ for the specialized SORule. For that, a naive method would enumerate all the possible intervals where the bounds are taken in $image(f_{l})$. However, the complexity is $O(\left| image\left( f_{l} \right) \right|^{2})$, that is not tractable for large sets of values. Thus, we employ the approach proposed by Fayyad and Irani [4] that filters out the values from $image(f_{l})$ that cannot be used as bounds in the best interval. This method is based on the values taken by the molecules in $coverage(d)$ that are associated with $q$ and those that are not. Intuitively, let us consider that $x_{1}\leq x_{2}\leq\ldots\leq x_{|image\left( f_{l} \right)|}$ the sorted set of values in $image(f_{l})$. The value $x_{i}$ is not the lower bound of the best interval if there exists $x_{j}$ with $j=i-1$ such that $\forall m\in coverage\left( d \right)$ where $m_{l}=x_{j}$, $q\subseteq class(m)$: Indeed, the lower bound $x_{j}$ for the interval leads to a better value for $f_{\beta}$ than the interval for which the lower bound is $x_{i}$. Similarly, the value $x_{i}$ is not the upper bound of the best interval if there exists $x_{j}$ with $j=i+1$ such that $\forall m\in coverage\left( d \right)$ where $m_{l}=x_{j}$, $q\subseteq class(m)$.

*Filtering out redundant rules.* We provide a similarity measure $sim$ that returns $true$ if two SORules are similar, $false$ otherwise. Basically, the similarity measure tests if the Jaccard coefficient [5] of the coverages of the two SORules are lower than a maximum similarity threshold $maxSim$. The redundancy filter works as follows: The set of SORules is sorted according to $F_{\beta}$ in a list $\Lambda$. Recursively, we poll (and remove) the best SORule $R$ from $\Lambda$, and we remove any SORule in $\Lambda$ that is similar to $R$. Note that in the experimental process, we set the maximum similarity threshold $maxSim$ to $0.6$ and the maximum number of output SORules *k* is set to 100 (MaxOutput), i.e., the algorithm returns the top 100 best diversified SORules. Eventually, we can neglect rules supported by too many odorant molecules with maxSupp parameter (*maxSupp* was set at 700 in our case).

**Supp Figure 1.** Description of the developed algorithm. (a) SORules search space with a beam search applied on panel “a”. (b) The beam search pseudo-code. (c) Illustration of the beam search-based exploration of the search space.

**References**

1. Kaytoue M, Kuznetsov SO, Napoli A. Revisiting numerical pattern mining with formal concept analysis. IJCAI Proceedings-International Joint Conference on Artificial Intelligence. 2011. p. 1342.

2. Lowerre BT. The HARPY speech recognition system. CARNEGIE-MELLON UNIV PITTSBURGH PA DEPT OF COMPUTER SCIENCE; 1976.

3. van Leeuwen M, Knobbe A. Diverse subgroup set discovery. Data Min Knowl Discov. 2012; 1–35.

4. Fayyad U, Irani K. Multi-interval discretization of continuous-valued attributes for classification learning. 1993;

5. Levandowsky M, Winter D. Distance between sets. Nature. 1971;234: 34.
